# Supplementary material for: Identification of side effects of COVID-19 drug candidates on embryogenesis using an integrated zebrafish screening platform
Source: Sci Rep. 2023 Oct 9;13:17037. doi: 10.1038/s41598-023-43911-3 (PMC10562458; doi:10.1038/s41598-023-43911-3)
Supplement: Supplementary file 1 — Supplementary Information. [file 41598_2023_43911_MOESM1_ESM.pdf]

Screening for side effects of COVID-19 drug candidates on cardiovascular development
Ernst, Piragyte et al., 2023

Supplementary Figures and Figure Legends

Figure S1

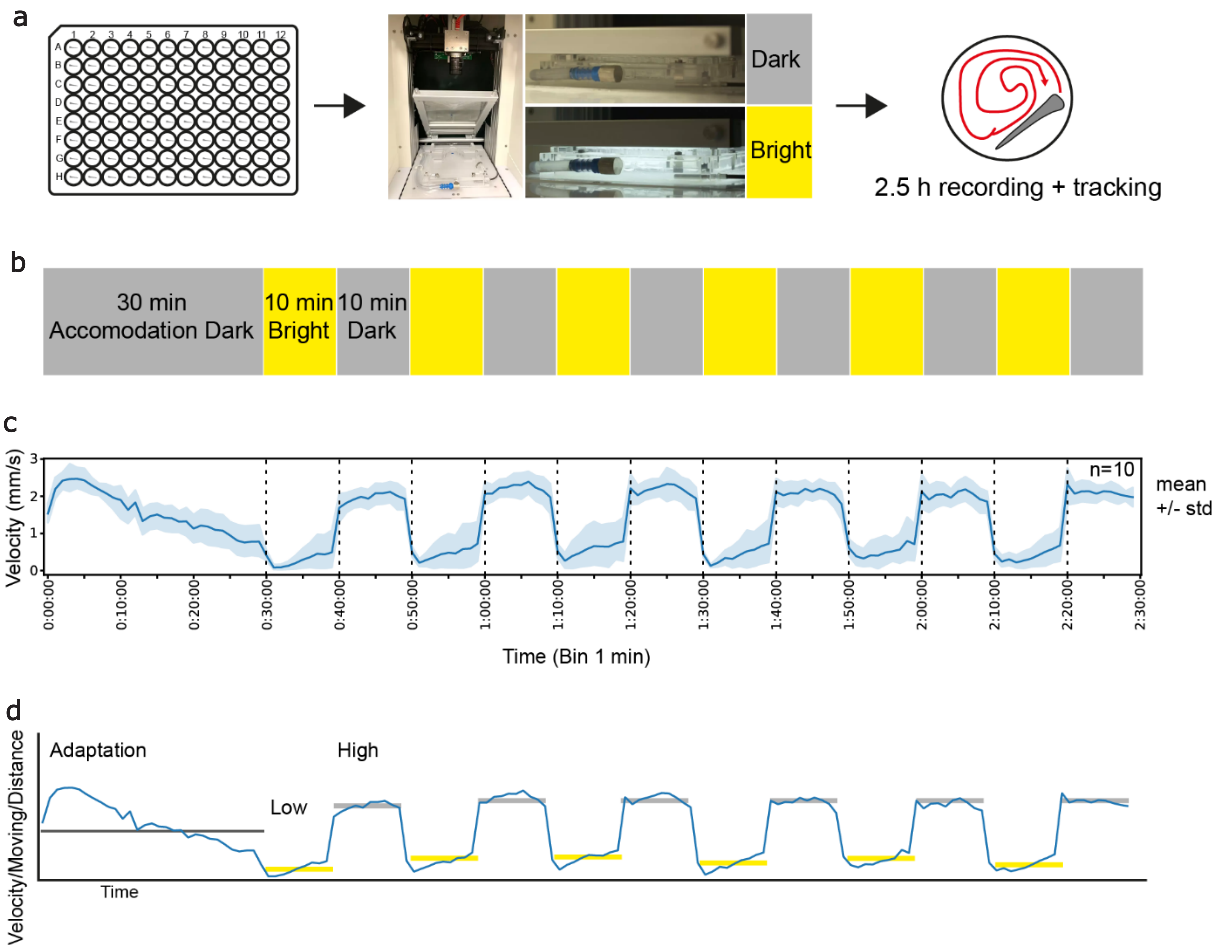

Screening for side effects of COVID-19 drug candidates on cardiovascular development
Ernst, Piragyte et al., 2023

Figure S2

	Pericardial Effusion Rate(%)	Mortality Bevavioural assay(%)	Mortality Morphological assay(%)	Mean Mortality(%)
(+)-Mefloquine_1.0	0.00	0.00	0.00	0.00
(-)-Anisomycin_1.0	12.50	0.00	0.00	0.00
(RS)-PPCC_1.0	11.11	0.00	0.00	0.00
ABT 239_1.0	11.76	5.00	0.00	2.50
AZ3451_1.0	25.00	0.00	0.00	0.00
Abacavir_1.0	0.00	0.00	0.00	0.00
Abemaciclib_1.0	0.00	0.00	0.00	0.00
Almitrine_1.0	0.00	0.00	0.00	0.00
Amiodarone_1.0	5.56	0.00	0.00	0.00
Amodiaquine_1.0	21.05	0.00	0.00	0.00
Amuvatinib_1.0	0.00	0.00	0.00	0.00
Anagliptin_1.0	0.00	5.00	0.00	2.50
Anidulafungin_1.0	0.00	0.00	0.00	0.00
**Apilimod_1.0**	88.89	100	0.00	50.00
Apixaban_1.0	0.00	0.00	0.00	0.00
Apremilast_1.0	0.00	10.00	0.00	5.00
Aprepitant_1.0	11.11	0.00	0.00	0.00
**Astemizole_1.0**	96.15	30.00	0.00	15.00
Atazanavir_1.0	0.00	0.00	0.00	0.00
Baricitinib_1.0	0.00	10.00	0.00	5.00
Bemcentinib_1.0	0.00	0.00	0.00	0.00
Benztropine_1.0	0.00	0.00	0.00	0.00
Berbamine_1.0	0.00	0.00	0.00	0.00
Boceprevir_1.0	0.00	0.00	0.00	0.00
Bortezomib_1.0	0.00	0.00	0.00	0.00
Brequinar_1.0	5.56	0.00	0.00	0.00
Cabozantinib_1.0	50.00	5.00	0.00	2.50
Camostat_1.0	0.00	0.00	0.00	0.00
Captopril_1.0	11.11	0.00	0.00	0.00
Celecoxib_1.0	0.00	10.00	0.00	5.00
Chlormidazole_1.0	33.33	0.00	0.00	0.00
Chlorothiazide_1.0	0.00	0.00	0.00	0.00
Chlorpromazine_1.0	0.00	0.00	0.00	0.00
Ciclesonide_1.0	60.00	0.00	0.00	0.00
Clemastine_1.0	10.00	0.00	0.00	0.00
Clomipramine_1.0	6.25	0.00	0.00	0.00
Cloperastine_1.0	12.50	0.00	0.00	0.00
Control_1.0	0.64	2.29	3.38	2.84
Cycloheximide_1.0	0.00	0.00	0.00	0.00
Cyclosporine_1.0	11.76	0.00	0.00	0.00
Dabrafenib_1.0	0.00	0.00	0.00	0.00
Daclatasvir_1.0	10.53	0.00	0.00	0.00
Danusertib_1.0	0.00	0.00	0.00	0.00
Dapivirine_1.0	0.00	0.00	0.00	0.00
Darifenacin_1.0	0.00	0.00	0.00	0.00
Darunavir_1.0	0.00	0.00	0.00	0.00
Delanzomib_1.0	0.00	0.00	0.00	0.00
Delavirdine_1.0	11.11	0.00	0.00	0.00
Desmethyl ferroquine_1.0	0.00	0.00	0.00	0.00
Digitoxin_1.0	0.00	0.00	5.00	2.50
Digoxin_1.0	5.56	0.00	0.00	0.00
Doravirine_1.0	25.00	0.00	0.00	0.00
Doxorubicin_1.0	0.00	0.00	0.00	0.00
Doxycycline_1.0	0.00	0.00	0.00	0.00

	Pericardial Effusion Rate(%)	Mortality Bevavioural assay(%)	Mortality Morphological assay(%)	Mean Mortality(%)
Drotaverine_1.0	11.11	0.00	0.00	0.00
Dutacatib_1.0	40.00	0.00	0.00	0.00
E 52862_1.0	0.00	0.00	0.00	0.00
Emetine_1.0	0.00	0.00	0.00	0.00
Entacapone_1.0	0.00	0.00	0.00	0.00
Eszopiclone_1.0	12.50	0.00	0.00	0.00
Ethaverine_1.0	0.00	0.00	0.00	0.00
Favipiravir_1.0	12.50	0.00	3.33	1.67
Ferroquine_1.0	0.00	0.00	0.00	0.00
Fluconazole_1.0	0.00	0.00	0.00	0.00
Fluphenazine_1.0	0.00	10.00	0.00	5.00
Fluspirilene_1.0	40.74	0.00	5.00	2.50
GSK 983_1.0	17.65	0.00	0.00	0.00
GSK-369796_1.0	5.56	0.00	0.00	0.00
Halofantrine_1.0	21.05	0.00	0.00	0.00
Haloperidol_1.0	11.11	0.00	0.00	0.00
Hanfangchin B_1.0	0.00	0.00	0.00	0.00
Hydroxychloroquine_1.0	11.11	0.00	0.00	0.00
Hydroxyprogesterone_1.0	16.67	0.00	0.00	0.00
Idelalisib_1.0	11.11	10.00	0.00	5.00
Imatinib_1.0	0.00	0.00	0.00	0.00
Indinavir_1.0	0.00	0.00	0.00	0.00
Indomethacin_1.0	14.29	0.00	0.00	0.00
Itraconazole_1.0	11.76	0.00	0.00	0.00
**Ivermectin_1.0**	5.56	100	0.00	50.00
JQ 1_1.0	11.11	0.00	0.00	0.00
Ketoconazole_1.0	0.00	0.00	0.00	0.00
LY 2228820_1.0	0.00	0.00	0.00	0.00
Lonafarnib_1.0	5.88	0.00	0.00	0.00
Lopinavir_1.0	0.00	0.00	0.00	0.00
Loratidine_1.0	0.00	0.00	0.00	0.00
Losartan_1.0	33.33	0.00	0.00	0.00
Lumefantrine_1.0	0.00	0.00	0.00	0.00
Lusutrombopag_1.0	0.00	0.00	0.00	0.00
MK-2206_1.0	0.00	0.00	10.00	5.00
MRT 68601_1.0	0.00	0.00	0.00	0.00
**Manidipine_1.0**	43.75	0.00	40.00	20.00
Merimepodib_1.0	0.00	0.00	0.00	0.00
Metformin_1.0	0.00	0.00	10.00	5.00
**Midostaurin_1.0**	35.29	45.00	10.00	27.50
Migalastat_1.0	0.00	0.00	0.00	0.00
Molnupiravir_1.0	5.00	0.00	0.00	0.00
Mycophenolic acid_1.0	0.00	0.00	0.00	0.00
N-Desethylamodiaquine_1.0	0.00	0.00	0.00	0.00
Nafamostat_1.0	0.00	0.00	0.00	0.00
Naphtoquine_1.0	12.50	10.00	0.00	5.00
Nebivolol_1.0	0.00	0.00	0.00	0.00
Nelfinavir_1.0	0.00	0.00	0.00	0.00
**Niclosamide_1.0**	No Data	100	100	100
Nifedipine_1.0	11.11	0.00	0.00	0.00
Nitazoxanide_1.0	0.00	0.00	0.00	0.00
ONO 5334_1.0	0.00	0.00	0.00	0.00
Osimertinib_1.0	22.22	0.00	0.00	0.00
Oxatomide_1.0	0.00	0.00	0.00	0.00

	Pericardial Effusion Rate(%)	Mortality Behavioural assay(%)	Mortality Morphological assay(%)	Mean Mortality(%)
Oxyclozanide_1.0	0.00	0.00	0.00	0.00
Ozanimod_1.0	25.00	0.00	0.00	0.00
PB 28_1.0	11.11	0.00	0.00	0.00
PD 144418_1.0	0.00	0.00	0.00	0.00
Papaverine_1.0	14.29	0.00	0.00	0.00
Paroxetine_1.0	0.00	0.00	0.00	0.00
Pevonedistat_1.0	0.00	0.00	0.00	0.00
Pexidartanib_1.0	0.00	0.00	0.00	0.00
**Pimozide_1.0**	100	25.00	0.00	12.50
Piperaquine_1.0	0.00	0.00	0.00	0.00
**Ponatinib_1.0**	100	0.00	0.00	0.00
Posaconazole_1.0	0.00	0.00	0.00	0.00
Promethazine_1.0	0.00	0.00	0.00	0.00
Proscillaridin_1.0	0.00	0.00	0.00	0.00
Pyronaridine_1.0	12.50	0.00	0.00	0.00
R 7112_1.0	0.00	0.00	0.00	0.00
RVX 208_1.0	0.00	0.00	0.00	0.00
Rapamycin_1.0	0.00	0.00	0.00	0.00
Ravuconazole_1.0	5.56	0.00	0.00	0.00
Regorafenib_1.0	58.82	0.00	0.00	0.00
Remdesivir_1.0	5.26	5.00	2.00	3.50
Ribavirin_1.0	6.25	0.00	0.00	0.00
Ritonavir_1.0	0.00	0.00	0.00	0.00
Ruxolitinib_1.0	0.00	0.00	0.00	0.00
SAX-187_1.0	0.00	0.00	0.00	0.00
SMN-C3_1.0	0.00	0.00	0.00	0.00
Salinomycin_1.0	0.00	0.00	0.00	0.00
Selumetinib_1.0	0.00	0.00	10.00	5.00
Sertindole_1.0	0.00	0.00	0.00	0.00
Silmitasertib_1.0	0.00	0.00	0.00	0.00
Simeprevir_1.0	6.25	0.00	5.00	2.50
Sofosbuvir_1.0	0.00	0.00	0.00	0.00
Sorafenib_1.0	88.89	0.00	0.00	0.00
Spectinomycin_1.0	0.00	0.00	0.00	0.00
Tacrolimus_1.0	31.58	5.00	5.00	5.00
Tenofovir_1.0	0.00	0.00	0.00	0.00
Terconazole_1.0	0.00	0.00	0.00	0.00
Tetracycline_1.0	0.00	0.00	20.00	10.00
Tetrandrine_1.0	0.00	0.00	0.00	0.00
Thiethylperazine_1.0	0.00	0.00	20.00	10.00
Tigecycline_1.0	0.00	0.00	0.00	0.00
Tioguanine_1.0	13.33	0.00	0.00	0.00
Tizoxanide_1.0	11.11	0.00	0.00	0.00
Tomivosertib_1.0	15.79	0.00	0.00	0.00
Toremifene_1.0	0.00	0.00	10.00	5.00
Triparanol_1.0	5.56	0.00	0.00	0.00
Umifenovir_1.0	23.53	0.00	5.00	2.50
Valdecoxib_1.0	0.00	0.00	0.00	0.00
Valproic Acid_1.0	5.56	0.00	0.00	0.00
Valsartan_1.0	0.00	0.00	0.00	0.00
Veliparib_1.0	0.00	0.00	0.00	0.00
Verapamil_1.0	0.00	0.00	0.00	0.00
Vidofludimus_1.0	7.69	0.00	0.00	0.00
ZINC4326719_1.0	0.00	0.00	10.00	5.00

% scale: 0 – 20 – 40 – 60 – 80 – 100; No Data

Figure S3

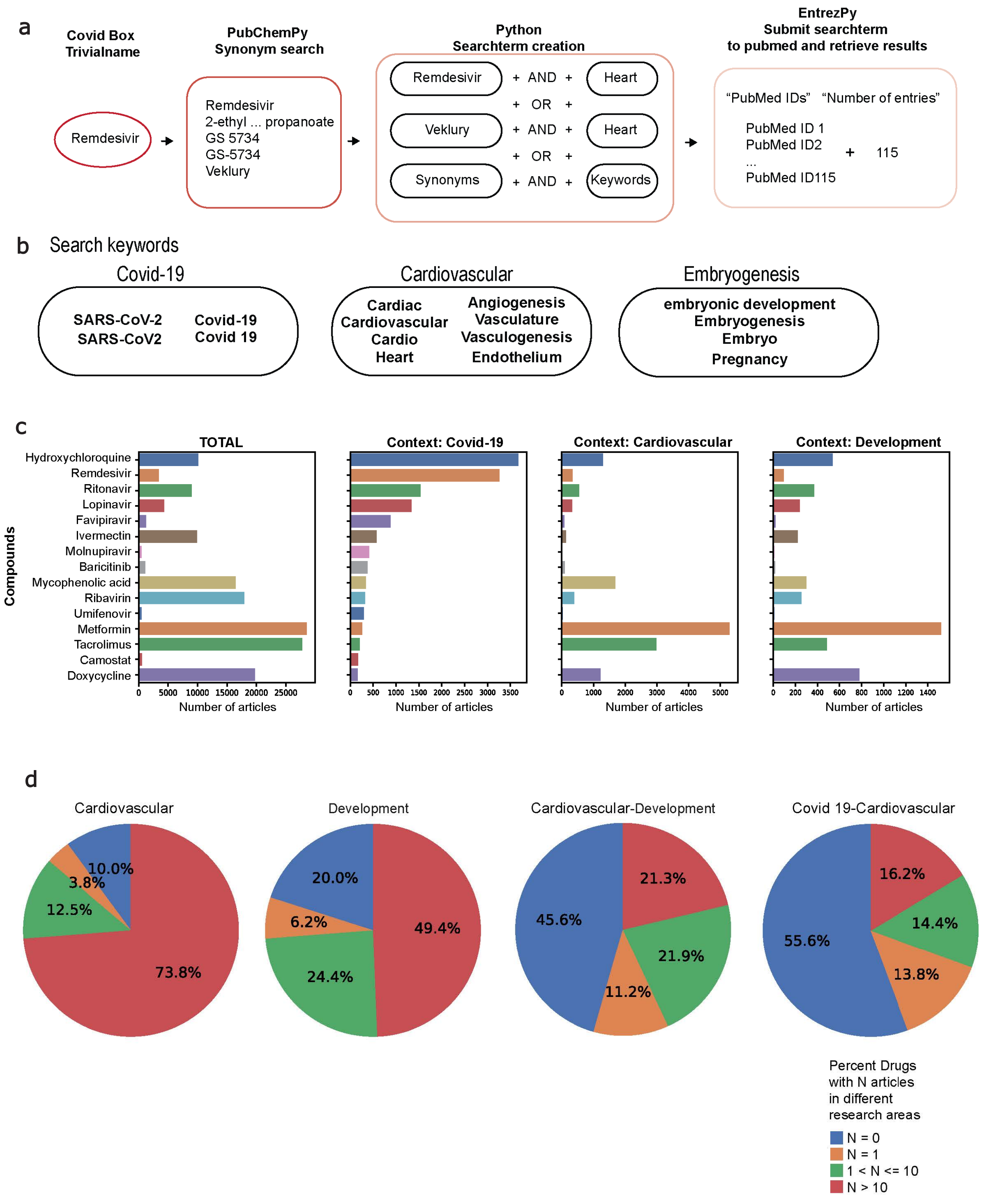
a
Covid Box
Trivialname
Remdesivir
PubChemPy
Synonym search
Remdesivir
2-ethyl ... propanoate
GS 5734
GS-5734
Veklury
Python
Searchterm creation
Remdesivir + AND + Heart
+ OR +
Veklury + AND + Heart
+ OR +
Synonyms + AND + Keywords
EntrezPy
Submit searchterm
to pubmed and retrieve results
"PubMed IDs" "Number of entries"
PubMed ID 1
PubMed ID2
...
PubMed ID115
+ 115
b Search keywords
Covid-19
SARS-CoV-2
SARS-CoV2
Covid-19
Covid 19
Cardiovascular
Cardiac
Cardiovascular
Cardio
Heart
Angiogenesis
Vasculature
Vasculogenesis
Endothelium
Embryogenesis
embryonic development
Embryogenesis
Embryo
Pregnancy
c
TOTAL
Context: Covid-19
Context: Cardiovascular
Context: Development
Compounds
Hydroxychloroquine
Remdesivir
Ritonavir
Lopinavir
Favipiravir
Ivermectin
Molnupiravir
Baricitinib
Mycophenolic acid
Ribavirin
Umifenovir
Metformin
Tacrolimus
Camostat
Doxycycline
0 5000 10000 15000 20000 25000
0 500 1000 1500 2000 2500 3000 3500
0 1000 2000 3000 4000 5000
0 200 400 600 800 1000 1200 1400
Number of articles
d
Cardiovascular
10.0%
3.8%
12.5%
73.8%
Development
20.0%
6.2%
24.4%
49.4%
Cardiovascular-Development
45.6%
21.3%
21.9%
11.2%
Covid 19-Cardiovascular
16.2%
14.4%
13.8%
55.6%
Percent Drugs
with N articles
in different
research areas
N = 0
N = 1
1 < N <= 10
N > 10

Screening for side effects of COVID-19 drug candidates on cardiovascular development
Ernst, Piragyte et al., 2023

Supplementary Figure Legends

Fig. S1. Workflow for behavioral studies in zebrafish larvae. a The free-swimming larvae in a 96 well-plate are transferred to the DanioVision recording chamber. The chamber can automatically switch on and off the illumination. Additionally, the larvae are recorded and tracked along 2.5 hours. **b** A bright-dark cycle is applied, starting with 30 min dark for accommodation. Then, the light is switched on for 10 min and switched off again for 10 min. This cycle is repeated in total 6 times. **c** A typical tracking result, here shown as velocity (mm/s) on the X-axis of 10 control larvae over time on the Y-axis (1 min binning, mean +/- standard deviation) is shown with the bright and dark phases (dashed lines). **d** For analysis, the programmed light cycle times are used. The control embryos typically swim less in the bright (yellow line) and more in the dark (grey line).

Fig. S2. Percentage Mortality and Pericardial Effusion in the drug screen. A heatmap showing Percentage of Pericardial effusion and Mortality in the drug screen. Mortality rates for Morphological Assay(with PTU) and Behavioural Assay (without PTU) are plotted separately followed by a column of mean mortality rate. Dark magenta shows the highest effect.

Fig. S3. Systematic literature search on published articles describing candidate compounds. a Schematic representation of how search term and results were retrieved. b In the first bar plot, the 15 compounds with the highest number of publications in the context of COVID-19 are shown. The drug names with black text color were the most mentioned in the literature in the context of COVID-19 plus Molnupiravir and Sabizabulin which was recently approved by several federal agencies for clinical treatment. The additional three plots contain the number of total articles, as well as articles on cardiovascular and embryogenesis research. c Pie charts showing the percentage of the compounds (n=162 drugs) with a defined threshold number of articles in the three contexts (Cardiovascular, Embryogenesis, Cardiovascular-Embryogenesis). The search was performed on the 20th of January 2023.
